# FGF14 Peptide Derivative Differentially Regulates Nav1.2 and Na_v_1.6 Function

**DOI:** 10.3390/life15091345

**Published:** 2025-08-25

**Authors:** Parsa Arman, Zahra Haghighijoo, Carmen A. Lupascu, Aditya K. Singh, Nana A. Goode, Timothy J. Baumgartner, Jully Singh, Yu Xue, Pingyuan Wang, Haiying Chen, Dinler A. Antunes, Marijn Lijffijt, Jia Zhou, Michele Migliore, Fernanda Laezza

**Affiliations:** 1 Department of Pharmacology & Toxicology, University of Texas Medical Branch, 301 University Blvd., Galveston, TX 77555, USA; parsaarman1@gmail.com (P.A.); haichen@utmb.edu (H.C.); jizhou@utmb.edu (J.Z.); 2 Institute of Biophysics, National Research Council, 90146 Palermo, Italy; 3 Department of Biology and Biochemistry, University of Houston, Houston, TX 77004, USA; 4 IonTX Inc., Friendswood, TX 77546, USA

**Keywords:** voltage-gated Na^+^ channel (Na_v_), automated planar patch electrophysiology, protein–protein interaction (PPI), FGF14, Nav1.6, Nav1.2, drug discovery, central nervous system (CNS)

## Abstract

Voltage-gated Na^+^ channels (Nav) are the molecular determinants of action potential initiation and propagation. Among the nine voltage-gated Na^+^ channel isoforms (Nav1.1–Nav1.9), Nav1.2 and Nav1.6 are of particular interest because of their developmental expression profile throughout the central nervous system (CNS) and their association with channelopathies. Although the α-subunit coded by each of the nine isoforms can sufficiently confer transient Na^+^ currents (I_Na_), in vivo these channels are modulated by auxiliary proteins like intracellular fibroblast growth factor (iFGFs) through protein–protein interaction (PPI), and probes developed from iFGF/Nav PPI complexes have been shown to precisely modulate Nav channels. Previous studies identified ZL0177, a peptidomimetic derived from a short peptide sequence at the FGF14/Nav1.6 PPI interface, as a functional modulator of Nav1.6-mediated I_Na_^+^. However, the isoform specificity, binding sites, and putative physiological impact of ZL0177 on neuronal excitability remain unexplored. Here, we used automated planar patch-clamp electrophysiology to assess ZL0177’s functional activity in cells stably expressing Nav1.2 or Nav1.6. While ZL0177 was found to suppress I_Na_ in both Nav1.2- and Nav1.6-expressing cells, ZL0177 elicited functionally divergent effects on channel kinetics that were isoform-specific and supported by differential docking of the compound to AlphaFold structures of the two channel isoforms. Computational modeling predicts that ZL0177 modulates Nav1.2 and Nav1.6 in an isoform-specific manner, eliciting phenotypically divergent effects on action potential discharge. Taken together, these results highlight the potential of PPI derivatives for isoform-specific regulation of Nav channels and the development of therapeutics for channelopathies.

## 1. Introduction

Voltage-gated Na^+^ (Nav; Nav1.1-Nav1.9) channels are the principal molecular determinants of the action potential [1,2,3,4]. Of particular interest, Nav1.2 (SCN2A) and Nav1.6 (SCN8A) are the predominant isoforms in principal neurons of the hippocampus, where they are enriched at the axonal initial segment (AIS) and contribute to the generation and propagation of the action potential [5,6,7]. During neurodevelopment, these channels undergo significant changes in expression and distribution, which contribute to their unique functional specialization in adulthood. At early neurodevelopmental stages, Nav1.2 is the most abundant isoform expressed at the AIS of principal neurons [8,9], but as maturation progresses, Nav1.6 replaces Nav1.2 [7]. With this developmental switch, Nav1.2 remains at the proximal AIS [10,11,12,13], aiding in action potential backpropagation and synaptic signal integration [8,9,10,11,13,14,15]. Conversely, Nav1.6 becomes dominant at the distal AIS, where it mediates forward action potential propagation [2]. Distinct expression patterns, developmental time courses, and subcellular localization of Nav1.2 and Nav1.6 lead to different outcomes for their pathological genetic variants [16,17,18]. SCN2A variants, mostly truncations, are associated with autism and intellectual disability [19,20], as well as epileptic encephalopathies [21], while SCN8A variants cause developmental and epileptic encephalopathies with motor symptoms [22,23,24,25]. This wide spectrum of disorders underscores the need for probing the Nav1.2 and Nav1.6 functional roles in healthy control conditions that could guide isoform-specific therapies for channelopathies.

The pore-forming α-subunit of Nav isoforms is sufficient to elicit Na^+^current in neurons, but their complete functionality relies on accessory proteins. Among those are the intracellular fibroblast growth factors (iFGFs), which interact with the Nav channel C-terminal tail domain (CTD) of various isoforms through protein–protein interactions (PPI). Each combination of iFGF and Nav channels forms a structurally unique complex that results in Na^+^ currents with distinct phenotypes. Consequently, the iFGF/Nav PPI interface has emerged as a promising target for developing probes to modulate excitability based on iFGF and Nav channel expression, structural features, and functional accessibility [12,26,27,28,29,30,31,32]. iFGF/Nav modulators either require both binding partners to be effective or can independently modulate Nav isoforms, likely by mimicking the iFGF interactor domain [30,33,34]. The latter group could serve as probes to study Nav isoform function independently of iFGF expression and aid in developing therapies for channelopathies.

We used HEK293 cells stably expressing only the pore-forming α-subunits of Nav1.2 or Nav1.6 to evaluate the isoform-selective effects of ZL0177, a previously characterized peptidomimetic derived from a structurally defined segment at the FGF14/Nav1.6 protein–protein interaction interface. Electrophysiological recordings were performed using automated planar patch clamp, and AlphaFold docking models were used to elucidate the binding modes of ZL0177 with Nav1.2 and Nav1.6. Additionally, computational modeling was done to assess the compound’s predicted impact on the divergent action potential profiles mediated by these isoforms in CA1 pyramidal neurons. Overall, the findings of this investigation demonstrate the value of using iFGF/Nav derivatives for isoform-specific modulation of Nav channels and their potential utility for the development of novel neurotherapeutics for channelopathies. ZL0177 is a peptidomimetic derived from the FLPK motif in the β12-sheet of FGF14 that mimics part of the FGF14/Nav interaction interface, but it is not intended to substitute for or displace endogenous FGF14.

## 2. Methods and Materials

### 2.1. Chemicals

ZL0177 was synthesized and provided by Dr. Jia Zhou’s Laboratory, with a molecular formula of C44H55N5O8 and a molecular weight of 781.95 g/mol. The compound quality was validated by NMR and HPLC analysis (purity > 99%) prior to the biological studies. Lu AE98134 was procured from MedChemExpress (Monmouth Junction, NJ, USA), with Catalog Number HY-133910 and CAS Number 849000-18-6. Tetrodotoxin Citrate was supplied by Bio-Techne Corporation (Tocris, Bristol, UK), with Catalog Number 1069. Extracellular and intracellular physiological and seal-enhancing solution were provided by Nanion Technologies (München, Germany).

### 2.2. Cell Culture

Human Embryonic Kidney 293 cells (HEK 293) stably expressing either SCN1A (coding for Na_v_1.1), SCN2A (coding for Na_v_1.2), or SCN8A (coding for Na_v_1.6) were used in this study and are referred to as Na_v_1.1, Na_v_1.2, and Na_v_1.6, respectively (gifted by Dr. Ortniz’s lab). They express only alpha subunit. The HEK cell lines used do not express detectable levels of FGF14, and no exogenous FGF14 was introduced. Additionally, these HEK lines do not express detectable levels of Nav β-subunits (SCN1B–SCN4B) and were not supplemented with exogenous β-subunit expression vectors. HEK293 cells were incubated at 37 °C and 5% CO2 in a 1:1 mixture of Dulbecco’s Modified Eagle Medium and F-12 supplemented with 10% fetal bovine serum, 100 units/mL of penicillin, and 100 µg/mL streptomycin. Growth medium was filtered for impurities using a sterile 90 mm diameter 0.22 µM PES CELLTREAT^®^ 500 mL filtration system prior to use. Prepared medium was then supplemented with additional antibiotics (0.5 mg/mL G418 and 5 μg/mL puromycin) to ensure continuous stable expression of Na_v_ isoforms. Cells were routinely checked to verify absence of mycoplasma contamination.

### 2.3. Automated Planar Hardware and Software

#### 2.3.1. Hardware

The porta-patch unit consists of the housing for the head stage, chip holder, Faraday lid, and the external/internal electrodes. This unit was used in conjunction with a Heka Patch Clamp EPC 10 amplifier. All internal and external electrodes are silver wires coated with silver chloride. The SuctionControl unit allows pressure (negative or positive) to be applied to the cell. It requires a silicone tube to be connected to the chip holder and suction control nozzle for calibration and experimentation.

#### 2.3.2. Software

PatchControl is a user interface software that consists of seven windows (Menu Bar, Experiment, PressureControl, Parameters, PerfusionControl, Macros, Log). PatchMaster (HEKA) is the electrophysiological software that consists of six windows (Amplifier, Replay, Oscilloscope, Control window, Notebook, Online window 1).

### 2.4. Automated Planar Patch Recordings Using the Porta-Patch

#### 2.4.1. Whole Cell Voltage-Clamp Solutions

HEK Cell Wash:

Dulbecco’s Phosphate-Buffered Saline (DPBS) 1X without Ca^2+^/Mg^2+^ was used to remove extracellular debris and non-functional cells prior to cell harvest, to increase the probability of higher quality cells reaching data acquisition.

HEK Cell Harvesting:

Gibco TrypLE^TM^ Express (without Phenol Red) was gently pipetted into the flask, which was then placed into an incubator at 37° C, 5% CO_2_ for 3–5 min to detach cells from the monolayer. After cell suspension, Gibco CHO-S-SFM II (without hypoxanthine or thymidine) was used to quench the enzymatic reaction prior to centrifugation. Cells were centrifuged at 1250 rpm for 4 min, and the supernatant was removed, leaving the cell pellet at the bottom. The cell pellet was then disaggregated in extracellular physiological solution.

Extracellular Physiological Solution:

The cell pellet was resuspended in an extracellular solution comprised of 140 mM NaCl, 4 mM KCl, 2 mM CaCl_2_, 1 mM MgCl_2_, 5 mM glucose, 10 mM HEPES, pH 7.3–7.4 (NaOH), 298 ± 3 mOsm. Cell suspension was transferred to a 4 °C chamber on top of a horizontal oscillator to maintain singularity during whole-cell patching. To note, this solution was placed on the external side of the borosilicate chip.

Intracellular Physiological Solution:

The solution placed in the interior portion of the borosilicate chip was comprised of 110 mM CsF, 10 mM NaCl, 10 mM CsCl, 10 mM EGTA, 10 mM HEPES, pH 7.2 (CsOH), 285 ± 3 mOsm.

External Seal Enhancing Solution:

The solution that was used to increase seal resistance (R_S_) prior to data acquisition was composed of 130 mM NaCl, 4 mM KCl, 1 mM MgCl_2_, 10 mM CaCl_2_, 5 mM D-glucose monohydrate, 10 mM HEPES, pH 7.4 (NaOH), 302 ± 3 mOsm. To note, this solution was swapped out with the external physiological solution before data acquisition to prevent the increased calcium from affecting the results.

#### 2.4.2. Automated Planar Cell Viability and Density Assessment

Prior to recording, HEK-293 cells were harvested using TrypLE™ Express Enzyme (1X) dissociation. TrypLE™ Express is singularized in external physiological solution. Cell viability was confirmed using Trypan Blue exclusion, and cell density was adjusted to approximately 1 × 10^6^ cells/mL to optimize capture efficiency. For whole-cell planar patch recordings, only cells that formed high-resistance seals (>500 MΩ) and exhibited inward Na^+^ currents ≥200 pA at −10 mV were included for analysis. Seal formation, series resistance, and capacitance were continuously monitored throughout the recordings using standard automated protocols.

Consistency in assay performance across cell lines (Nav1.1, Nav1.2, Nav1.6) was maintained by following standardized preparation protocols, and no significant variation in basic electrophysiological parameters was observed between recording sessions. All inclusion criteria used for voltage-gated sodium channel analysis are further detailed in Section 2.6. Exclusion thresholds were defined based on quality control recommendations from the instrument manufacturer (Nanion Technologies), supplemented by internal benchmarking. Cells were excluded from analysis if they exhibited seal resistance <500 MΩ, series resistance >20 MΩ, or leak current exceeding ±40 pA, to ensure recording fidelity and minimize biophysical variability.

### 2.5. Automated Patch-Clamp Recordings Using the Synchropatch 384i

Cells were recorded in whole-cell mode using the Synchropatch 384i (Nanion Technologies, Munich, Germany). Electrophysiological protocols were constructed, and data were acquired using PatchControl 384 and DataControl 384 software (Nanion Technologies, Munich, Germany).

Prior to recording, HEK-Nav1.1, HEK-Nav1.2, or HEK-Nav1.6 cells were exposed to vehicle (0.1% DMSO) or increasing concentrations of compound ZL0177 in External Standard Solution (Cat# 08 3001, Nanion Technologies) for 30 min. After the incubation period, internal solution (Cat# 08 3008, Nanion Technologies) was loaded into the intracellular compartment of a 384-well Nanion patch clamp (NPC) chip (Nanion Technologies). Next, Extra Fill solution (Cat# 08 3003) was loaded into NPC chip wells (30 μL/well). HEK-Nav1.1, Nav1.2, or Nav1.6 (human embryonic kidney) cell suspension (at least 400,000 cells/mL in External Standard solution supplemented with DMSO or 1063) was pipetted into NPC wells (20 μL/well). Catch (−150 mbar for 5 s) and hold (−50 mbar for 30 s) pressures were applied to enrich cell capture. Seal Enhancer solution (External Standard with 13mM CaCl2 and 6.5mM MgCl2) was then added to NPC wells (40 μL/well). Fifty microliters per well was then removed, and NPC wells were washed with External Standard (40 μL/well). Forty microliters per well was then removed from each NPC well before proceeding to whole-cell configuration. Throughout seal formation and over the course of recordings, electrophysiological parameters such as seal resistance, capacitance, and series resistance were monitored using Patchcontrol 384.

Initial APC data observation and analysis were conducted using Datacontrol 384. Cells selected for analysis displayed a seal resistance of at least 0.5 GΩ and minimum baseline current of −200pA. Sweep data for cells meeting inclusion criteria were exported and tabulated in Microsoft Excel, and data were analyzed using GraphPad Prism version 10.

### 2.6. Voltage-Clamp Data Analysis

Current densities (pA/pF) were obtained by dividing Na^+^ current (I_Na_) amplitude by membrane capacitance (C_fast_). Current–voltage (IV) relationships were then assessed by plotting current density (pA/pF) as a function of the applied voltage (mV). Peak current densities were obtained from −10 mV of applied voltage. To assess voltage dependence of activation, conductance (G_Na_) was first calculated using the following equation:

G_Na_ = I_Na_/(V_m_ − E_rev_)

where I_Na_ is the current amplitude at voltage V_m_, and E_rev_ is the Na^+^ reversal potential (60 mV). Activation curves were then generated by plotting normalized G_Na_ as a function of the test potential. Data were then fitted with the Boltzmann equation to determine V_1/2_ of steady-state activation using the following equation:

G_Na_/G_Na,Max_ = 1 + *e*^(Va − Em)/k^

where G_Na,Max_ is the maximum conductance, V_a_ is the membrane potential of half-maximal activation, E_m_ is the membrane voltage, and k is the slope factor. For steady-state inactivation, the normalized current amplitude (I_Na_/I_Na,Max_) at the test potential was plotted as a function of the pre-pulse potential (V_m_) and fitted using the Boltzmann equation:

I_Na_/I_Na,Max_ = 1/1 + *e*^(Vh − Em)/k^

where V_h_ is the potential of half-maximal inactivation, E_m_ is the membrane voltage, and k is the slope factor.

The voltage error (V_Error_) was calculated using the equation [35]:

$$V_{\mathrm{Error}}\;=\;[\mathrm{Peak}\;\mathrm{Current}\;\mathrm{at}\;-\;10\;\mathrm{mV}\;(\mathrm{nA})]\cdot R_{U}\cdot (1-R_{\mathrm{Comp}})$$

where R_U_ is the access resistance and R_Comp_ is the fraction series resistance compensation.

The Z-factor for peak I_Na_ density at −10 mV was calculated using the equation below.


$$Z’=1-\{[(3\cdot {\mathrm{SD}}_{\mathrm{Max}})+(3\cdot {\mathrm{SD}}_{\mathrm{Min}})]/{\mathrm{Mean}}_{\mathrm{Max}}-{\mathrm{Mean}}_{\mathrm{Min}})\}$$

#### Nav1.2 and Nav1.6 Structure Prediction and Ligand Docking Studies

All structural modeling and docking analyses were performed using full-length human Nav1.2 (UniProtKB: Q99250) and Nav1.6 (UniProtKB: Q9UQD0) sequences to ensure consistency in residue numbering and relevance to human pathophysiology.

Initially, both Na_v_1.2 (UniProtKB AC:Q99250) and Na_v_1.6 ( UniProtKB AC: Q9UQD0) AlphaFold (AF) models were fitted to the transmembrane X-ray crystallographic structure of Na_v_1.2 (PDB ID: 6J8E), cryo-EM of Na_v_1.6 (PDB ID: 8FHD), and X-ray crystal structure of Na_v_1.2’s C-terminal domain (PDB ID: 4JPZ) using ChimeraX (UCSF, San Francisco, CA, USA). In addition, the AF models were assessed manually using stereochemical analysis tools within COOT (Crystallographic Object-Oriented Toolkit; MRC Laboratory of Molecular Biology, Cambridge, UK). Additionally, to enhance the accuracy of the docking process, the structure quality analysis of the predicted models was assessed using PROCHECK and ProSA- web server. Furthermore, the AF-modeled structures were preprocessed using the OPLS4 force field within Epik’s Protein Preparation Wizard Tool (PPWT) on Schrödinger Small-Molecule Drug Discovery Suite (Schrödinger, New York, NY, USA). The SiteMap, a geometry-based pocket-finding method, was utilized to identify potential binding sites for ligand docking. The minimum number of site points was set, and the output was limited to five sites. Those five sites were reduced using cytoplasmic regions only. Additionally, a more restrictive hydrophobicity definition and 4 Å nearest-point site crop method were employed. Grid generation using Glide was carried out on predicted cytoplasmic sites, with the grid center coordinates set to X = −14.60, Y = −14.78, Z = 24.77 for Na_v_1.2 and X = −18.04, Y = −17.31, Z = 25.31 for Na_v_1.6. The grid box size was set to 20 Å on each side. The 3D structure of ZL0177 was generated in Schrödinger Maestro, and a low-energy conformation was computed using LigPrep. Ligand docking was performed using Glide with the standard precision (SP) protocol. In Schrödinger Maestro, the top-scoring docked poses with the lowest ΔG (kcal/mol) values were selected and visualized for ligand–protein interactions.

### 2.7. Computational CA1 Pyramidal Neuron Modeling

All simulations were carried out using the NEURON simulator v8.0.0 (Yale, New Haven, CT, USA) [36]. Model and simulation files are available in ModelDB “https://modeldb.science/2014826 (accessed on 19 August 2025)”.

The basic computational model was composed of a single compartment (5 μm diameter and length) with passive properties consistent with those measured in CA1 pyramidal neurons (C_m_ = 1 μF/cm^2^, R_m_ = 30,000 Ω/cm^2^), with a resting potential set to −70 mV. Temperature was fixed at 20 °C. In addition to the appropriate Na^+^ channel isoform, a delayed rectifier (K_DR_) and K_v_7 (K_M_) channel were added—taken from a previously published realistic model of CA1 pyramidal neurons—to study the number of action potentials elicited as a function of the input current [37]. The equations describing each channel’s kinetics are described elsewhere [38]. Here we report those for the sodium channel:


$$I_{\mathrm{Na}}\;=\;G_{\mathrm{Na}}\cdot m^{3}\cdot h\cdot (v-E_{\mathrm{rev}})$$

$$dm/dt=(m_{\infty }-m)/\tau_{m}$$

$$dh/dt=(h_{\infty }-h)/\tau_{h}$$

$$m_{\infty }=a_{m}/(a_{m}+b_{m})$$

$$\tau_{m}=0.7579/(a_{m}+b_{m})$$

$$a_{m}=R_{a}\cdot (v-{𝒱}_{Act,50})/[1-\exp\{-(v-{𝒱}_{Act,50})/q_{a}\}]$$

$$b_{m}=-R_{b}\cdot (v-{𝒱}_{Act,50})/[1-\exp\{(v-{𝒱}_{Act,50})/q_{a}\}]$$

$$h_{\infty }=1/[1+\exp\{(v-{𝒱}_{Inact,50})/q_{h}\}]$$

$$\tau_{h}=0.7579/(a_{h}+b_{h})$$

$$a_{h}=R_{d}\cdot (v-{\mathrm{thi}}_{1})/[1-\exp\{-(v-{\mathrm{thi}}_{1})/q_{d}\}]$$

$$b_{h}=-R_{g}\cdot (v-{\mathrm{thi}}_{1})/[1-\exp\{(v-{\mathrm{thi}}_{1})/q_{d}\}]$$

*v*—membrane potential.

*m*—activation curve.

*h*—inactivation curve.

G_Na_—peak conductance.

E_rev_—Na^+^ reversal potential.

*τ*_*m*_—activation time constant.

*τ*_*h*_—inactivation time constant.

a*_h_*/b_*h*_—rate constant for inactivation.

a_*m*_/b_*m*_—rate constant for activation.

𝒱_Act,50_—half-point of activation.

𝒱_Inact,50_—half-point of inactivation.

thi_1_—half-point of time constant for inactivation.

*q*_a/_*q*_d/_*q*_*h*_—shape factors.

R_a_/R_b_/R_d_/R_g_—scaling factor where V_1/2_ of activation was set to selected values from representative cells from each group: −22.3 mV for Na_v_1.6 DMSO, −9.73 for Na_v_1.6 10 μM ZL0177, −25.1 for Na_v_1.2 DMSO, and −29.0 for Na_v_1.2 10 μM ZL0177. Similarly, V_1/2_ of inactivation was set to −58.3 for Na_v_1.6 DMSO, −51.9 for Na_v_1.6 10 μM ZL0177, −55.3 for Na_v_1.2 DMSO, and −61.9 for Na_v_1.2 10 μM ZL0177. The Na^+^ channel E_rev_ was extracted from experimental findings (+50 mV for Na_v_1.6 and +60 mV for Na_v_1.2).

The raw electrophysiological current traces from one representative cell for each condition were used as reference to implement the sodium kinetics under the different conditions. We selected cell 5 for Na_v_1.6 DMSO, cell 5 for Na_v_1.6 10 μM ZL0177, cell 3 for Na_v_1.2 DMSO, and cell 6 for Na_v_1.2 10 μM ZL0177. Traces recorded under different voltage-clamp conditions were simultaneously fitted using the built-in Multiple Run Fitter NEURON tool. The midpoints of activation and inactivation curves, but not their slopes, were fixed to the average experimental values. See main text for final values of the other model parameters used for fitting.

### 2.8. Statistical Analysis of Data

Statistical analysis was performed using GraphPad Prism. All datasets were first tested for normality using the Shapiro-Wilk test, and homogeneity of variances was assessed using Levene’s test. When both assumptions were met, parametric analyses were applied. Specifically, one-way ANOVA followed by Tukey’s multiple comparisons test was used for comparing peak current density, V_1_/_2_ of activation, and V_1_/_2_ of inactivation. In cases where assumptions of normality or equal variance were violated, appropriate non-parametric tests were used. For paired comparisons, two-tailed paired t-tests were applied. All data are reported as mean ± SD. Statistical details, including test type, *N* values, and *p*-values, are included in the figure legends or main text.

## 3. Results

### 3.1. ZL0177 Modulates Na^+^ Currents in an Isoform-Specific Manner

**Figure 1:**
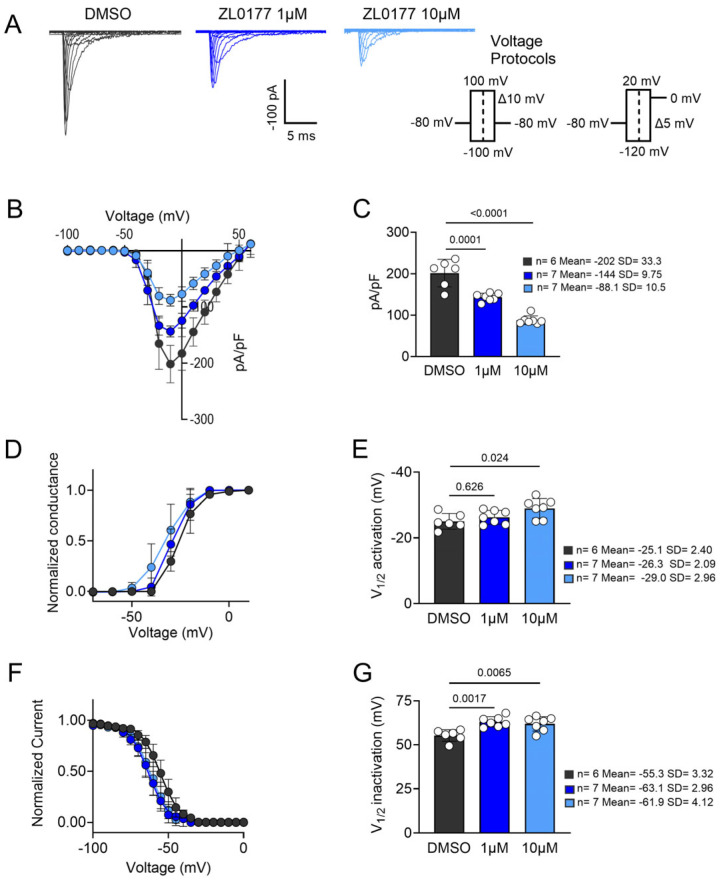
**Pharmacological characterization of ZL0177 on Na_v_1.2-mediated currents.** (**A**) Average peak I_Na_-matched representative traces for stably expressing HEK-Na_v_1.2 cells are shown for control (0.1% DMSO, black) and treated groups [(ZL0177 1 µM (dark blue), 10 µM (light blue)] with the current density elicited at −10 mV. (**B**) Current (pA/pF) voltage (mV) relationship is shown from −60 mV to +60 mV (Δ 10 mV) for control (*n* = 6) and treated groups (*n* = 7). (**C**) Bar graphs representing peak I_Na_ density at −10 mV for both control and treated groups are shown. (**D**) Normalized conductance plotted as a function of voltage to characterize the effects of ZL0177 on the voltage-dependence of activation of Nav1.2 in all three experimental groups. Plotted data were fitted with the Boltzmann equation to determine V_1/2_ of activation. (**E**) V_1/2_ of activation derived from the plot in D. (**F**) Normalized current plotted as a function of voltage for all three experimental groups to characterize the effects of ZL0177 on the voltage-dependence of steady-state inactivation. Plotted data were fitted with the Boltzmann equation to determine V_1/2_ of inactivation. (**G**) Comparison of V_1/2_ of steady-state inactivation from all three experimental groups. One-way ANOVA with post-hoc Tukey’s comparisons was conducted to determine statistical significance. All data shown reflect recordings from HEK cells expressing only the human Nav α-subunit (Nav1.2 or Nav1.6), without β-subunits. Data are presented as mean ± SD.

In previous studies, we demonstrated that a single concentration of ZL0177 (10 µM) suppresses Nav1.6-mediated I_Na_ and causes a depolarizing shift in the V_1/2_ of activation [29]. However, the isoform selectivity of the compound has yet to be determined. In this study, we focused on comparing the effects of ZL0177 on Nav1.2 and Nav1.6 channels, which have unique specializations in neurons and age-dependent expression patterns. To compare ZL0177’s activity on Nav1.2 and Nav1.6 currents, HEK-293 cells stably expressing either Na_v_1.2 or Nav1.6 were incubated with either ZL0177 (1 µM or 10 µM) or 0.1% DMSO in growth medium for 1 h. After processing, cells were subjected to two voltage-clamp protocols, allowing for transient I_Na_ activation and steady-state inactivation kinetics to be collected. Our observations for Nav1.2 are summarized in Figure 1. Representative traces (Figure 1A) and accompanying current-voltage relationship (IV) curves (Figure 1B) are displayed. The peak (−10 mV) I_Na_ density (pA/pF) shows a statistically significant reduction at 1 µM ZL0177 (*p* = 0.0001; *n* = 7; Mean = −144; SD = 9.75) and at 10 µM ZL0177 (*p* < 0.0001; *n* = 7; Mean = −88.1; SD = 10.5) when compared with 0.1% DMSO (*n* = 6; Mean = −202; SD = 33.3; Figure 1C). A representative Boltzmann-fitted voltage dependence of the activation plot is shown in Figure 1D. Further analysis of activation kinetics revealed a statistically significant hyperpolarizing shift in the V_1/2_ of activation for Nav1.2 at 10 µM (*p* = 0.0242; *n* = 7; Mean = −29.0; SD = 2.96), but not at 1 µM (*p* = 0.626; *n* = 7; Mean = −26.3; SD = 2.09), when compared with 0.1% DMSO (*n* = 6; Mean = −25.1; SD = 2.40; Figure 1E). A representative Boltzmann-fitted voltage dependence of the steady-state inactivation plot is shown in Figure 1F. Analysis of inactivation kinetics indicated a statistically significant hyperpolarizing shift in the V_1/2_ of inactivation for Nav1.2 at both 1 µM (*p* = 0.00170; *n* = 7; Mean = −63.1; SD = 2.96) and 10 µM (*p* = 0.00650; *n* = 7; Mean = −61.9; SD = 4.12) when compared with 0.1% DMSO (*n* = 6; Mean = −55.3; SD = 3.32; Figure 1G).

**Figure 2:**
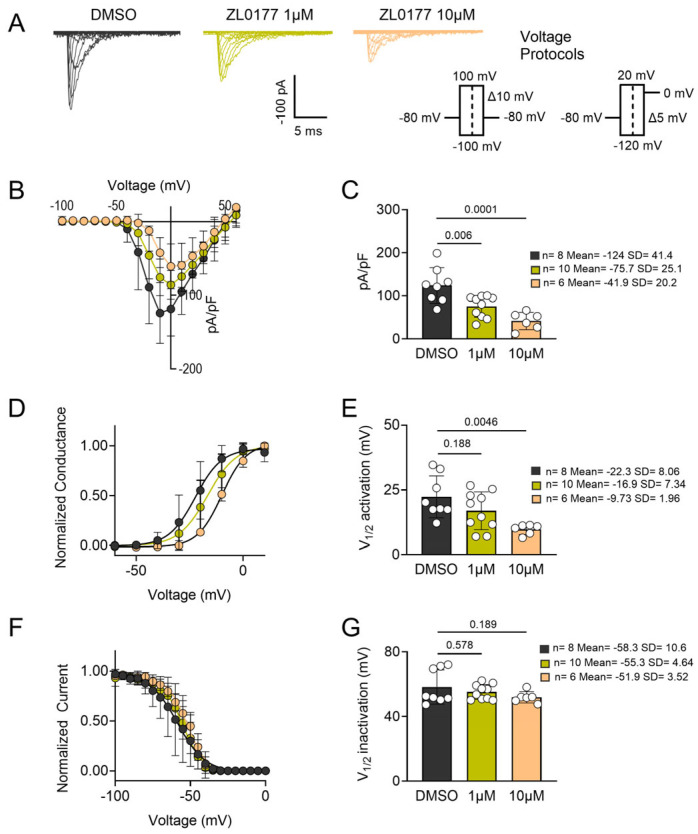
**Pharmacological characterization of ZL0177 on Nav1.6-mediated currents.** (**A**) Average peak I_Na_-matched representative traces for stably expressing HEK-Na_v_1.6 cells are shown for control (0.1% DMSO, black) and treated groups [ZL0177 1 µM (avocado), 10 µM (peach)] with the current density elicited at −10 mV. (**B**) Current (pA/pF) voltage (mV) relationship is shown from −60 mV to +60 mV (Δ 10 mV) for control (*n* = 6) and treated groups (*n* = 7). (**C**) Bar graphs representing peak I_Na_ density at −10 mV for both control and treated groups are shown. (**D**) Normalized conductance plotted as a function of voltage to characterize the effects of ZL0177 on the voltage-dependence of activation of Nav1.6 in all three experimental groups. Plotted data were fitted with the Boltzmann equation to determine V_1/2_ of activation. (**E**) V_1/2_ of activation derived from the plot in D. (**F**) Normalized current plotted as a function of voltage for all three experimental groups to characterize the effects of ZL0177 on the voltage-dependence of steady-state inactivation. Plotted data were fitted with the Boltzmann equation to determine V_1/2_ of inactivation. (**G**) Comparison of V_1/2_ of steady-state inactivation from all three experimental groups. One-way ANOVA with post-hoc Tukey’s multiple comparisons was conducted to determine statistical significance. All data shown reflect recordings from HEK cells expressing only the human Nav α-subunit (Nav1.2 or Nav1.6), without β-subunits. Data are presented as mean ± SD.

Next, we evaluated the impact of ZL0177 on Nav1.6-mediated currents using the same concentrations as for Nav1.2. This extended our initial findings and allowed for a direct comparison with Nav1.2. The results for Nav1.6 are presented in Figure 2. We observed a significant reduction in Nav1.6-mediated peak (−10 mV) INa density at both 1 µM ZL0177 (*p* = 0.00600; *n* = 10; Mean = −75.7; SD = 25.1) and 10 µM ZL0177 (*p* = 0.000100; *n* = 6; Mean = −41.9; SD = 20.2) compared with 0.1% DMSO (*n* = 8; Mean = −124; SD = 41.4; Figure 2C). A Boltzmann-fitted voltage-dependence activation plot is shown in Figure 2D. Analysis of Nav1.6 activation kinetics revealed no significant depolarizing shift in the V_1/2_ of activation at 1 µM (*p* = 0.188; *n* = 10; Mean = −16.9; SD = 7.34), but a significant depolarizing shift was observed at 10 µM ZL0177 (*p* = 0.00460; *n* = 6; Mean = −9.73; SD = 1.96) compared with 0.1% DMSO (*n* = 8; Mean = −22.3; SD = 8.06; Figure 2E). However, ZL0177 did not affect the voltage dependence of steady-state inactivation at either lower or higher concentrations, as shown in Figure 2F. Inactivation kinetics analysis showed no significant change in the V_1/2_ of inactivation for Nav1.6 at either 1 µM (*p* = 0.578; *n* = 10; Mean = −55.3; SD = 4.64) or 10 µM (*p* = 0.189; *n* = 6; Mean = −51.9; SD = 3.52) compared with 0.1% DMSO (*n* = 8; Mean = −58.3; SD = 10.6; Figure 2G). The observed phenotypes for Nav1.6 are consistent with those observed previously following treatment with ZL0177 [29].

To supplement the observed functional effects of ZL0177 on Nav1.2 and Nav1.6-mediated currents, additional studies were conducted using the Synchropatch 384i (Table 1). In agreement with the initial experiment, we observed a significant suppression of Nav1.2-mediated currents following treatment with 10 μM ZL0177 (−228.3 ± 133.3; *n* = 22) compared with vehicle control (0.1% DMSO) (−130.7 ± 92.8; *n* = 17; *p* = 0.0143). Regarding Nav1.6, we observed a suppression of peak current density following treatment with ZL0177 (−21.6 ± 7.7; *n* = 20) compared with vehicle control (−51.2 ± 28.2; *n* = 17; *p* < 0.0001). These findings are congruent with the results for Nav1.6 observed using the porta-patch.

Overall, these results indicate that despite the uniform effect of ZL0177 on the amplitude of I_Na_ mediated by Nav1.2 or Nav1.6, the compound exhibits isoform-specific modulation of channel voltage dependence. Notable differences include the direction of the shift in V_1/2_ of activation (Nav1.2 shows hyperpolarization, while Nav1.6 shows depolarization) and the lack of effect of the compound on the regulation of inactivation in Nav1.6, contrasted with a strong hyperpolarizing effect on Nav1.2.

### 3.2. AlphaFold Structure of Nav1.2 and Nav1.6 Channels and Predicted ZL0177 Binding Sites

**Figure 3:**
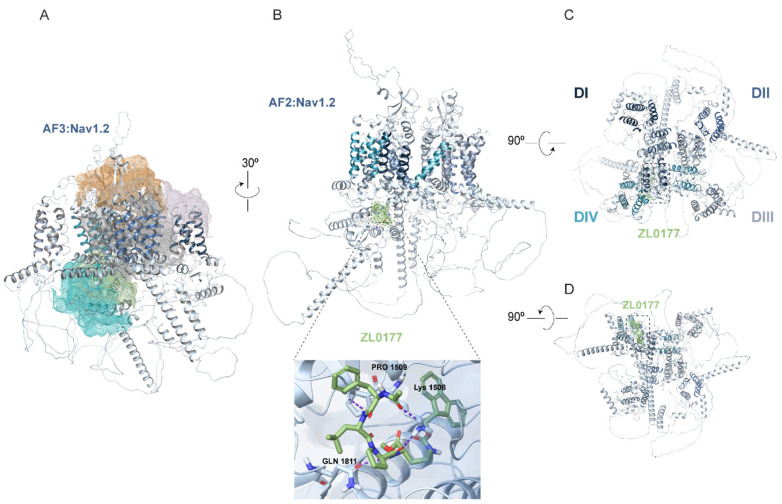
**AF structure of the Nav1.2 and predicted ZL0177 binding site.** (**A**) Ribbon representation of the side view of the human Nav1.2 α subunit of the channel, with colors corresponding to the structural components of the DI-IV domains. The transmembrane segments (S1–S6) for each domain are depicted in shades of blue. The predicted druggable pockets are shown as mesh surfaces: orange, pink, and gray highlight the druggable pockets in the pore and voltage-sensing domains, while green and turquoise indicate the cytoplasmic domains. (**B**) Illustrates the molecular docking result of the predicted AF2 structure of Nav1.2 and the ligand ZL0177. Ribbon representation of the side view of the α subunit of the Nav1.2 channel, in shades of blue corresponding to the structural components. The predicted active site is shown as a green surface. The dotted box highlights the specific region, shown magnified in a close-up view, detailing the predicted binding site and hydrogen bond interactions (purple dots) of ZL0177 with Lys1508, Pro1509, and Gln1811. (**C**) A representative top view of Nav1.2 is displayed, showing the overall structure from an overhead perspective. (**D**) A representative bottom view of Nav1.2 is presented, providing a view of the structure from underneath. Residue numbering is based on the human Nav1.2 sequence (UniProtKB: Q99250).

**Figure 4:**
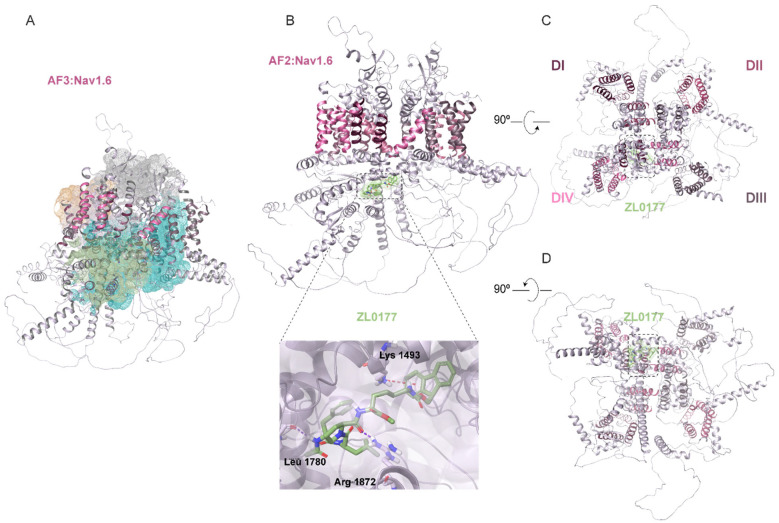
**AF structure of the Nav1.6 and predicted ZL0177 binding site.** (**A**) Ribbon representation of the side view of the human Nav1.6 α subunit of the channel, with colors corresponding to the structural components of the DI-IV domains. The transmembrane segments (S1–S6) for each domain are depicted in shades of pink. The predicted druggable pockets are shown as mesh surfaces: orange, pink, and gray highlight the druggable pockets in the pore and voltage-sensing domains, while green and turquoise indicate the cytoplasmic domains. (**B**) Illustrates the molecular docking result of the predicted AF structure of Nav1.6 and the ligand ZL0177. Ribbon representation of the side view of the α subunit of the Nav1.6 channel is shown in shades of pink corresponding to the structural components. The predicted active site is depicted as a green surface. The dotted box highlights a specific region, shown magnified in a close-up view, revealing two hydrogen bond (H-bond) interactions (purple dots) of ZL0177 with Leu1780 and Arg1872. Additionally, ZL0177 interacts with Lys1493 via a single π-cation bond (red dots). (**C**) A representative top view of Nav1.6 is displayed, showing the overall structure from an overhead perspective. (**D**) A representative bottom view of Nav1.6 is presented, providing a view of the structure from underneath. Residue numbering is based on the human Nav1.6 sequence (UniProtKB: Q9UQD0).

To gain insights into the isoform-specific activity of ZL0177 on Nav1.2 and Nav1.6 channels, computational investigations using AF were conducted to dock and visualize the putative binding sites of ZL0177 on Nav1.2 and Nav1.6. Since the full-length 3D crystallographic structures of Nav1.2 and Nav1.6 are currently unavailable, the quality of our AF modeling was assessed using segmented cryo-EM and X-ray crystallographic structures. The primary sequence similarity between Nav1.2 and Nav1.6 is 75.42%. Therefore, the predicted AF models of Nav1.2 and Nav1.6 were superimposed onto the cryo-EM structure of Nav1.2 (PDB ID: 6J8E) for the Cα atoms belonging to the transmembrane (TM) α helices (aa 1-1777), achieving backbone RMSD values of 0.899 Å and 0.744 Å, respectively. Additionally, the C-terminal X-ray crystallographic structure (aa 1789-1929) of Nav1.2 (PDB ID: 4JPZ) was fitted to both models using ChimeraX, with RMSD values of 1.417 Å and 1.363 Å. The superimposed AF_Nav1.6 model onto the cryo-EM structure of Nav1.6 (PDB ID: 8FHD) yielded an RMSD value of 0.935 Å. Next, the Sitemap tool was applied to determine the binding sites of the predicted model for Nav1.2. Based on scores and overall surface area analysis, five potential druggable pockets were predicted (colored mesh surfaces, Figure 3A), with the cytoplasmic region surrounding the inactivation gate (amino acids 1470-1532) and the proximal CTD (amino acids 1776-1895) emerging as the top-ranked site (green and turquoise mesh surfaces, Figure 3A). This region contains a plausible surface area of H-bond acceptor/donor and hydrophobic sites. The docking results obtained with AF2 show that ZL0177 interacts with Lys1508, Pro1509, and Gln1811 of Nav1.2 (Figure 3B). ZL0177 primarily interacts with Lys1508 via two hydrogen bonds. Additionally, it interacts with Pro1509 via one hydrogen bond and with Gln1811 via another hydrogen bond. These amino acids are part of the inactivation gate (Lys1508 and Pro1509) and the proximal CTD (Gln1811) of Nav1.2. Representative top-down (Figure 3C) and bottom-up (Figure 3D) views indicate that ZL0177 interacts with cytoplasmic regions of Nav1.2. Based on the AF structure prediction, Sitemap also revealed five potential druggable pockets on Nav1.6 (Figure 4A). The docking analysis showed significant interactions between ZL0177 and Lys1493, Leu1780, and Arg1872 (Figure 4B). ZL0177 predominantly forms hydrogen bonds with Leu1780 and Arg1872, with one bond per amino acid, indicating a strong interaction with the distal CTD (amino acids 1766-1980). ZL0177 also engages with residue Lys1493, situated within the inactivation gate, through a weaker π-cation bond, which aligns with the lack of activity of ZL0177 on steady-state inactivation. Similar to Nav1.2, representative top-down (Figure 4C) and bottom-up (Figure 4D) views indicate that ZL0177 interacts with cytoplasmic regions of Nav1.6. Overall, the AF-based docking predictions supported the electrophysiological findings regarding ZL0177’s activity on Nav1.2 and Nav1.6 currents. They indicated stronger and more involved interactions for ZL0177 with Nav1.2, mediated by three H-bonds on the CTD and inactivation gate, compared with Nav1.6, which showed two H-bonds on the CTD and a weak π-cation bond on the inactivation gate.

### 3.3. Zl0177 Elicits Phenotypically Divergent Action Potentials in CA1 Pyramidal Neuron Computational Models

Next, we sought to explore the potential effect of ZL0177 on a computational model of CA1 pyramidal neurons in which only one of the two Nav isoforms was the predominant isoform. This computational configuration allowed us to predict the impact that ZL0177 may have in vivo in subcellular compartments of CA1 neurons, where either one of the Nav isoforms is enriched, or in different developmental stages during which the two isoforms are differentially expressed.

**Figure 5:**
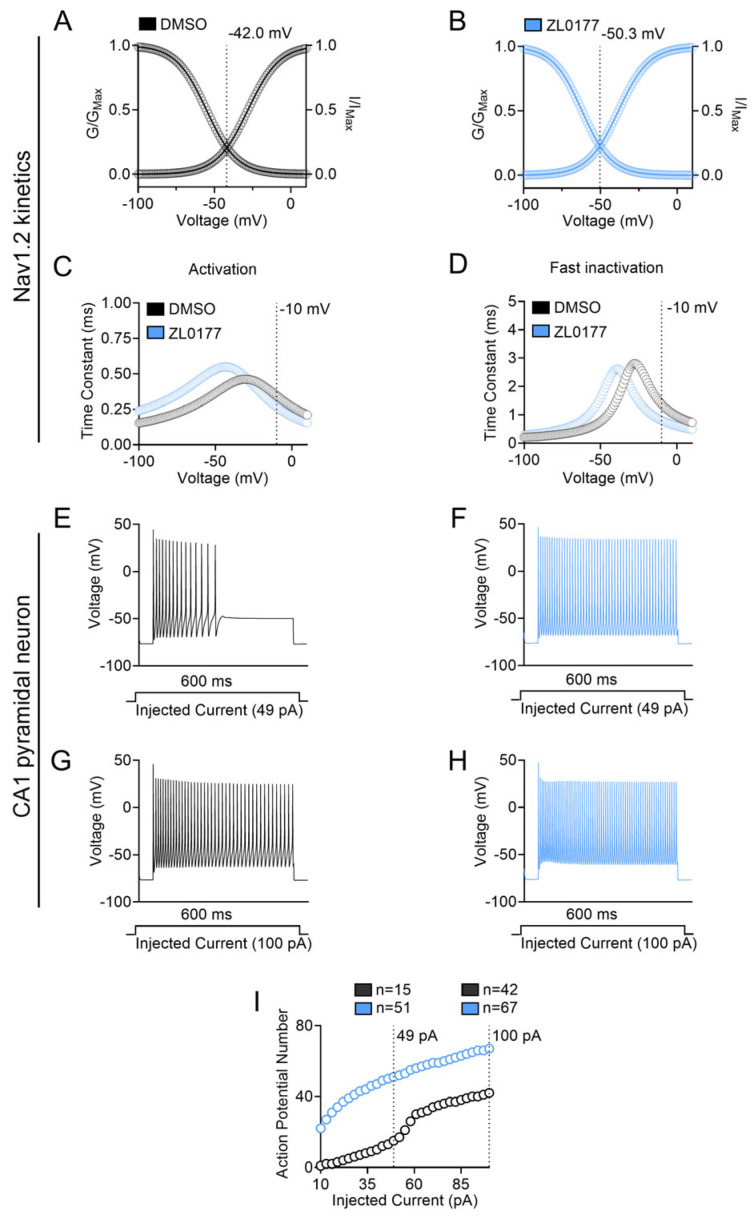
**Computational model of Na_v_1.2 kinetics and CA1 pyramidal neuron firing**. (**A**) While using values obtained experimentally (−25.1 mV for Na_v_1.2 0.1% DMSO V_1/2_ of activation and −55.3 mV for Na_v_1.2 0.1% DMSO V_1/2_ of inactivation) from Na_v_1.2 cell 3, all other models’rameters were simultaneously fitted using the built-in Multiple Run Fitter NEURON v8.0.0; DMSO (black) and ZL0177 (light blue). (**B**) While using values obtained experimentally (−29.0 mV for Na_v_1.2 10 μM V_1/2_ of activation and −61.9 mV for Na_v_1.2 10 μM V_1/2_ of inactivation), Na_v_1.2 cell 6’s all other models’rameters were simultaneously fitted using NEURON. (**C**) Activation time constants plotted as a function of voltage (mV) for Na_v_1.2 control and 10 µM ZL0177. (**D**) Inactivation time constants plotted as function of voltage (mV) for Na_v_1.2 control and 10 µM ZL0177. (**E**) Representative train of action potentials elicited by a 600 ms 49 pA injected current for control. (**F**) Representative train of action potentials elicited by a 600 ms 49 pA injected current for 10 µM ZL0177. (**G**) Representative train of action potentials elicited by a 600 ms 100 pA injected current for control. (**H**) Representative train of action potentials elicited by a 600 ms 100 pA injected current for 10 µM ZL0177. (**I**) Action potential frequency shown as a function of injected current from 10 pA to 100 pA for both control and 10 µM ZL0177.

First, the voltage-clamp kinetics of Nav1.2 activation and inactivation under control (0.1% DMSO) and treated (10 µM ZL0177) conditions were computed (Figure 5). Using experimentally obtained values (−25.1 mV for V_1/2_ of activation and −55.3 mV for V_1/2_ of inactivation in DMSO, and −29.0 mV for V_1/2_ of activation and −61.9 mV for V_1/2_ of inactivation in ZL0177), all other model’s parameters were simultaneously fitted using the built-in Multiple Run Fitter NEURON v8.0.0. The simulated kinetics predicted a hyperpolarizing shift in window current for Nav1.2 in the presence of 10 µM ZL0177 compared with 0.1% DMSO control. The intersection between steady-state activation and steady-state inactivation for 0.1% DMSO and 10 µM ZL0177 occurred at approximately −42 mV and −50.3 mV, respectively (Figure 5A,B). Additionally, ZL0177 caused a generalized hyperpolarizing shift in the time constant of steady-state activation and inactivation (Figure 5C,D). At −10 mV, the time constant of steady-state activation for 0.1% DMSO and 10 µM ZL0177 was approximately 0.347 ms and 0.266 ms, respectively (Figure 5C). At −10 mV, the time constant of steady-state inactivation for 0.1% DMSO and 10 µM ZL0177 was approximately 1.38 ms and 0.805 ms, respectively (Figure 5D). Next, a single-compartment neuron model including Nav1.2, delayed rectifier (K_DR_), and K_v_7 (K_M_) channels, along with passive properties that mimic CA1 pyramidal neurons, was built, and current-clamp simulations were carried out using the NEURON simulator. In this model, the number of action potentials elicited was recorded during a 600 ms long square input-current injection step, ranging from 10 to 100 pA, under 0.1% DMSO and 10 µM ZL0177 conditions. At 49 pA of injected current, the application of 10 µM ZL0177 (51 action potentials) increased the number of action potentials compared with 0.1% DMSO controls (15 action potentials) (Figure 5E,F). Furthermore, at 100 pA of injected current, the application of 10 µM ZL0177 (67 action potentials) increased the number of action potentials compared with 0.1% DMSO controls (42 action potentials) (Figure 5G,H). In conclusion, at both 49 pA and 100 pA, the number of action potentials elicited as a function of injected current increased compared with the controls (Figure 5I).

**Figure 6:**
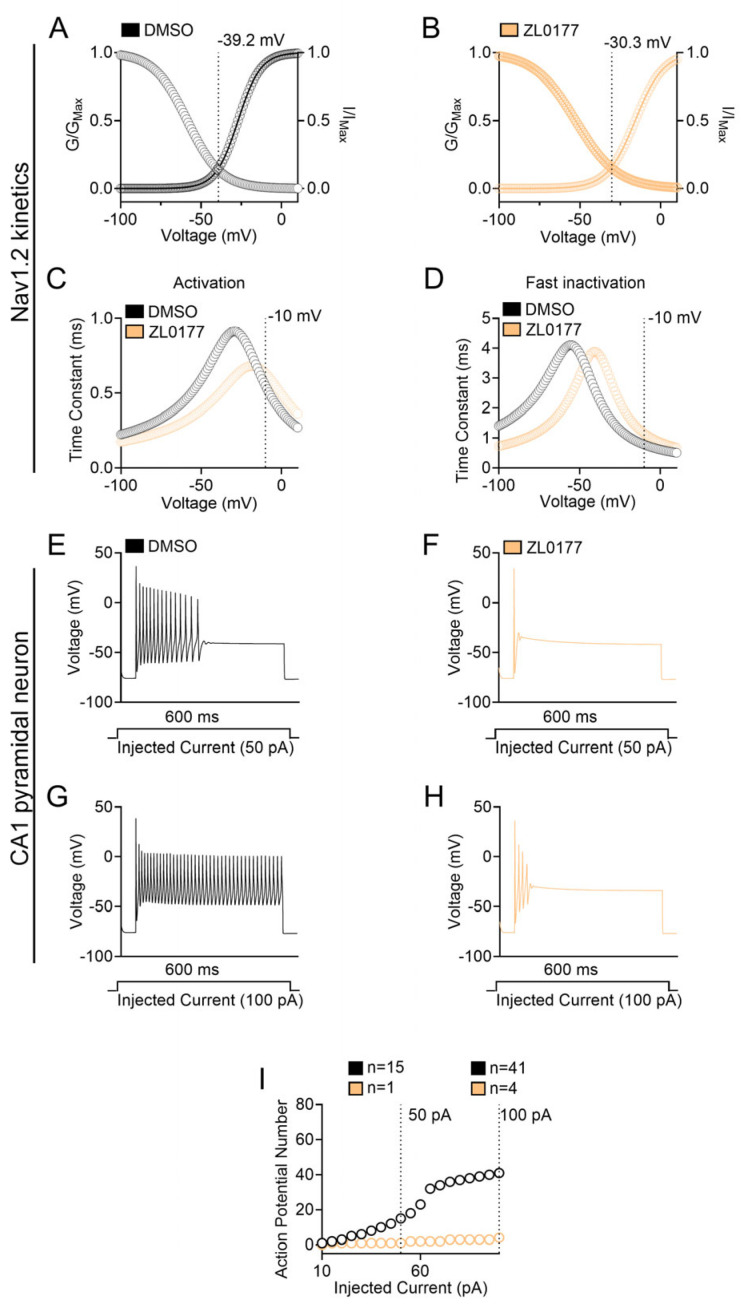
**Computational Model of Na_v_1.6 kinetics and CA1 Pyramidal Neuron firing**. (**A**) While using values obtained experimentally (−22.3 mV for Nav1.6 0.1% DMSO V_1/2_ of activation and −58.3 mV for Nav1.6 0.1% DMSO V_1/2_ of inactivation), Nav1.6 cell 5’s and all other models’parameters were simultaneously fitted using the built-in Multiple Run Fitter NEURON tool; DMSO (black) and ZL0177 (peach). (**B**) While using values obtained experimentally (−9.73 mV for Nav1.6 10 μM V_1/2_ of activation and −51.9 mV for Nav1.6 10 μM V_1/2_ of inactivation) from Nav1.6 cell 5, all other models’parameters were simultaneously fitted using the built-in Multiple Run Fitter NEURON tool. (**C**) Activation time constants plotted as a function of voltage (mV) for Nav1.6 0.1% DMSO and 10 µM ZL0177. (**D**) Inactivation time constants plotted as a function of voltage (mV) for Nav1.6 0.1% DMSO and 10 µM ZL0177. (**E**). Representative train of action potentials elicited by a 600 ms 49 pA injected current for 0.1% DMSO. (**F**) Representative train of action potentials elicited by a 600 ms 49 pA injected current for 10 µM ZL0177. (**G**) Representative train of action potentials elicited by a 600 ms 100 pA injected current for 0.1% DMSO. (**H**) Representative train of action potentials elicited by a 600 ms 100 pA injected current for 10 µM ZL0177. (**I**) Action potential frequency shown as a function of injected current from 10 pA to 100 pA for both 0.1% DMSO and 10 µM ZL0177.

Corresponding simulations were computed for Na_v_1.6 kinetics and a current-clamp CA1 pyramidal model under control (0.1% DMSO) and treated (10 µM ZL0177) conditions (Figure 6). In the case of Nav1.6, the intersection between steady-state activation and steady-state inactivation for 0.1% DMSO and 10 µM ZL0177 occurred at approximately −39.2 mV and −30.3 mV, respectively (Figure 6A,B). Additionally, ZL0177 caused a generalized depolarizing shift in the time constant of steady-state activation and inactivation (Figure 6C,D). At −10 mV, the time constants of steady-state activation for 0.1% DMSO and 10 µM ZL0177 were approximately 0.548 ms and 0.632 ms, respectively (Figure 6C). At −10 mV, the time constants of steady-state inactivation for 0.1% DMSO and 10 µM ZL0177 were approximately 0.783 ms and 1.16 ms, respectively (Figure 6D). The current clamp recordings in the respective CA1 pyramidal neuron model, including Na_v_1.6 as the only source of Na^+^ currents, showed a remarkable efficacy of ZL0177 in decreasing the number of action potentials when compared with 0.1% DMSO, resulting in a complete suppression of intrinsic firing at any injection steps (Figure 6E–I). These results corroborate an isoform-specific activity of ZL0177 on Nav channels and suggest an opposite effect on phenotypically divergent action potentials elicited by Nav1.2 or Nav1.6.

## 4. Discussion

Here, we expanded upon previous work on peptide derivatives of hot-spot residues at the FGF14/Nav1.6 PPI [30,33,34] interface by evaluating the functional effects of ZL0177 on Nav1.2 and Nav1.6. These isoforms are of particular interest due to their biological function at the AIS within pyramidal neurons [2,5,39,40,41,42] and implication in numerous channelopathies [18,19,20,21,22,23,24,25]. Nav1.2 and Nav1.6 precisely coordinate the forward and back propagation of action potentials (APs) in pyramidal neurons [5,6], and the individual contributions of Nav1.2 and Nav1.6 to these processes are ascribed to their unique membrane expression profiles. In mature neurons, Nav1.6 is the predominant isoform expressed in the distal AIS, which is the site of AP initiation, and facilitates forward propagation of APs through the axon [2]. Nav1.2 exhibits a higher relative density distribution in the proximal AIS, lending to its role in facilitating back propagation of APs to the soma and dendrites [2,5]. During development, Nav1.2 is the predominant isoform expressed throughout the AIS of pyramidal neurons [6,7], where it is later replaced by Nav1.6 in the mature brain [1,43]. Loss-of-function (LOF) mutations to the SCN2A gene, encoding Nav1.2, are associated with increased rates of autism spectrum disorder [44].

Despite its AIS enrichment during early development, SCN2A LOF mutations induce pathological neuronal hyperexcitability [45], which is thought to be an artifact of decreased dendritic potassium channel recruitment, perturbing neuronal repolarization [6,45]. In the mature brain, SCN8A (Nav1.6) gain-of-function mutations cause hyperexcitability in pyramidal neurons, resulting in epilepsy [46,47,48], while LOF mutations suppress neuronal excitability and are implicated in intellectual disabilities, cognitive deficits, and motor impairment [48,49,50,51]. These age-dependent functional roles and causal disease linkages position Nav1.2 and Nav1.6 as attractive molecular targets for various neuropsychiatric and neurodevelopmental disorders.

Given the unique functional roles of Nav1.2 and Nav1.6 in regulating the AP, isoform specificity is an essential aspect of therapeutic development. The pursuit of molecules to selectively modulate Nav1.2 or Nav1.6 is particularly arduous on account of their high degree of structural homology [52,53]. An alternative approach to direct pharmacological targeting of Nav channels is through allosteric modulation of their regulatory PPIs [53]. Nav channels are functionally modulated through numerous intracellular PPIs, which alter channel gating properties and elicit the full range of Nav channels’ physiological functions [26,28,29,30,31,32,54]. Among these intracellular binding partners are iFGFs, which have been observed to induce unique and divergent isoform-specific functional effects on Nav channel activity [15,27,28,29,31,32,54,55,56,57,58,59,60]. Thus, targeting specific iFGF/Nav pair complexes with unique tissue expression and subcellular localization profiles is a promising approach for developing probes that can precisely modulate Na^+^ currents and neuronal firing in distinct brain regions. Peptides or small molecules derived from iFGF/Nav pathological phenotypes associated with Nav channel dysfunction [31,32,55]. Pairs can either modulate these complexes (i.e., PPI modulators) or act independently, functioning as mimics of iFGFs. This diversity provides a range of options for modulating different Nav channel isoforms, potentially enabling precise control of I_Na_ currents. Previous manual patch-clamp electrophysiological studies show that treatment with 10 μM ZL0177 suppressed Nav1.6-mediated currents and induced a depolarizing shift in the V_1/2_ of activation without significantly impacting steady-state inactivation [29]. In the present work, we built upon initial experiments and observed the same effects resulting from treatment with 10 μM ZL0177 using two automated patch-clamp modalities.

We used both the porta-patch and SynchroPatch 384i automated patch-clamp (APC) platforms. The porta-patch system was used for detailed kinetic analyses due to its precise control over voltage protocols, while the SynchroPatch 384i was leveraged for higher-throughput screening of current densities. Notably, we observed differences in baseline current densities across the two platforms, particularly for Nav1.6, likely due to differences in seal quality, chip properties, or cell access resistance. These variations are consistent with prior reports comparing APC systems and emphasize the importance of interpreting current amplitude data within the technical context of the platform used. Nonetheless, key findings such as suppression of peak current by ZL0177 were replicated across both systems, reinforcing the robustness of our observations.

Functional evaluation of ZL0177 in HEK-Nav1.2 cells revealed a suppression of Nav1.2-mediated currents, similar to that observed in Nav1.6. However, divergent effects on channel kinetics were observed between the two isoforms: ZL0177 induced a hyperpolarizing shift in both the V_1/2_ of activation and the V_1/2_ of steady-state inactivation of Nav1.2-mediated currents, in contrast to the depolarizing shift seen in Nav1.6. Notably, all V_1/2_ measurements were obtained using the porta-patch system, as the SynchroPatch was used solely for current density validation due to its higher throughput.

ZL0177 is a tetrapeptide derived from FLPK, a sequence within the β12 sheet of FGF14 involved in interactions with Nav1.6 [31]. Specific modifications to enhance its properties were introduced to FLPK. An acetyl functional group was added to the N-terminal phenylalanine moiety, while an Fmoc function and methoxy groups were linked to the lysine residue at the C-terminus of the tetrapeptide. These modifications, combined with divergence in the amino acid sequence of the two Nav channel isoforms, potentially contribute to the unique functional activity of ZL177 in modulating Nav1.2 and Nav1.6 channels.

The potential structural basis for the isoform-specific effects of ZL0177 was explored using AF models. These models revealed that ZL0177 interacts with specific domains of Nav1.2 and Nav1.6 through hydrogen bonds and π-cation bonds. The predicted druggable pockets surrounding the inactivation gate, formed by the intracellular loop linking the DIII-S6 and DIV-S1 domains, and the CTD of Nav1.2, correspond to potential binding sites for ZL0177. These two active sites (Figure 3A, mesh surface in orange and grey) correspond to previously identified binding sites of natural toxins and/or therapeutic drugs within other Nav channel isoforms [61], providing a reference template for our study. Specifically, ZL0177 was found to interact with the proximal CTD (Gln1811) and inactivation gate (Lys1508 and Pro1509) of Nav1.2 through H-bonds (Figure 3) between the acetyl group on the N-terminal and specific residues in the inactivation domain and CTD. Conversely, ZL0177 was found to interact strongly with the Nav1.6 distal CTD (Leu1780 and Arg1872) through hydrogen bonds and weakly with the inactivation gate (Lys1493) through π-cation bonds (Figure 4). Notably, this cytoplasmic CTD region was ranked highest among the five predicted binding pockets for Nav1.6 based on interaction energy and clustering scores, reinforcing its potential as a biologically relevant docking site. These interactions appear to involve the phenylalanine moiety and the Fmoc protecting group at the N-terminus interacting with residues in the inactivation domain and CTD.

Notably, ZL0177 binds within intracellular cytoplasmic pockets distinct from the classical pore or voltage-sensor domains typically targeted by natural toxins or drugs. These canonical sites were used as docking templates to identify and exclude structurally conserved regions likely to yield non-specific interactions across Nav isoforms. ZL0177 is not structurally related to these compounds. Instead, its predicted interaction with the C-terminal domain (CTD), particularly near the FGF14 β12-strand binding motif, supports a mechanism of selective modulation consistent with its design. This suggests that CTD-adjacent pockets may serve as allosteric sites capable of isoform-specific regulation, potentially informing future therapeutic strategies for channelopathies.

Under non-depolarizing conditions, most Nav channels are in the resting closed state. The transition from closed to open state requires strong depolarization, enabling the S4 segments within the DI-DIV domains to quickly move outward, driving the opening of the activation gate. Following this conformational change, the inactivation particle, a cytoplasmic IFMT motif linking DIII and DIV, causes the channel to transition from the open state to the fast-inactivated state [62,63,64,65,66]. Nav channels can also proceed to the inactivated state from the closed state, giving rise to steady-state—also known as closed-state—inactivation. In these conformational changes, the voltage sensitivity is orchestrated by multiple regions, including the cytoplasmic linkers connecting the segments of S4–S5 in DIII and DIV, as well as the proximal cytoplasmic end of S6 in DIV [63]. Thus, it is plausible that ZL0177 interacts with one or multiple S4–S5 loops of Nav1.2, strongly associating with inactivation binding sites while sterically hindering the pore, which would account for the modulations in peak current density and steady-state inactivation. For Nav1.6, ZL0177 might similarly interact with one or multiple S4–S5 loops and sterically hinder the pore, explaining its modulation of peak current density and activation. In contrast to Nav1.2, the weak interactions with the inactivation binding sites of Nav1.6 could explain the lack of effect on Nav1.6 inactivation. Ultimately, while the precise molecular underpinnings conferring the directional divergence of ZL0177’s effects on Nav1.2 and Nav1.6 channel kinetics are unclear, it is likely a result of its distinct intermolecular interactions with their respective CTDs. As ZL0177 binds intracellular sites, future protocols could include supplementing the internal recording solution to mitigate potential compound washout during whole-cell configuration and improve signal stability.

To reconcile differences with prior studies reporting peptidomimetic binding to Nav1.6, we note that our docking approach relied on full-length AlphaFold2-predicted human Nav1.6 models, refined against available cryo-EM and crystallographic structures. Additionally, ZL0177 differs in sequence and structure from previously described peptides derived from the FGF14 interface, which may further account for distinct binding interactions. Future comparative docking or mutagenesis studies will be valuable to clarify these differences.

There are limitations in predicting these interactions using AF. First, Nav channels, being complex membrane proteins, present challenges for structural determination due to their size, membrane-bound nature, and dynamic behavior. Experimental protein structures for the full-length Nav1.2 and Nav1.6 channels are limited to structures for specific domains or regions, such as the voltage-sensing domains (VSDs) or pore domains, in complex with auxiliary subunits or interacting proteins. However, the lack of experimentally resolved structures of these two Nav channel isoforms is a limitation. Second, predictions through AF can offer valuable insights. However, the ability of AF to generate biologically accurate intracellular loops when predicting differences in ZL0177 activity regarding activation, inactivation, and peak current density is limited due to the disordered structure of some Nav channel segments. Despite these limitations, the docking results obtained are meaningful and consistent with our experimental findings. Specifically, ZL0177 formed hydrogen bonds with Nav1.2 domains associated with activation, inactivation, and regulation of peak current density. Conversely, ZL0177 exhibited weak interactions (pi–pi) with the Nav1.6 inactivation gate, aligning with its lack of effect on Nav1.6 inactivation.

To complement these in silico predictions with human relevance, we explored whether naturally occurring pathogenic variants may support the functional significance of the predicted ZL0177 binding residues. We queried publicly available human genetic variant databases (e.g., ClinVar, HGMD) for disease-linked missense mutations affecting key residues identified in our docking models, including Lys1508, Pro1509, and Gln1811 in Nav1.2, and Lys1493, Leu1780, and Arg1872 in Nav1.6. While we identified pathogenic variants in the broader CTD region (e.g., Arg1882Gln in Nav1.2 and Asn1768Asp in Nav1.6), none overlapped with the specific residues predicted to interact with ZL0177. These results suggest that, although the binding regions are structurally plausible, further mutagenesis or patient-variant analyses will be required in the future to confirm their functional relevance.

Building on these structural and genetic insights, results from the computational modeling predict that ZL0177 will have differential effects on neuronal firing mediated by Nav1.2 or Nav1.6. In line with the predicted effect of the compound on the respective window current mediated by Nav1.2 or Nav1.6, the model predicts that ZL0177 would increase action potential firing mediated by Nav1.2 while suppressing action potential firing mediated by Nav1.6. These effects could culminate in differing functional outcomes depending on the neurodevelopmental stage of neurons. During early development, when Nav1.2 is the most abundantly expressed Nav isoform in the AIS [42], ZL0177 could increase the overall excitability of the neuron with impacts on synapse formation and maturation [67]. By contrast, in mature principal neurons, Nav1.2 is restricted to the proximal AIS [2,7,44], and Nav1.6 is the predominant isoform in the distal AIS [2,42]. Thus, in mature neurons, ZL0177 could enhance somato-dendritic potentials and synaptic integration via modulation of Nav1.2 [2,67] while suppressing forward propagation of the action potential through functional downregulation of Nav1.6 [1,6].

While this study focused on Nav1.2 and Nav1.6 due to their defined roles in excitatory pyramidal neurons and established FGF14 interaction profiles, Nav1.1 is also a major isoform in the brain, particularly in inhibitory interneurons. Although initial screening included Nav1.1, we did not pursue follow-up analyses of its gating properties or ZL0177 sensitivity in this study. However, evaluating whether ZL0177 modulates Nav1.1 is essential to understanding its broader effects on neuronal circuit excitability, particularly regarding potential disinhibition. Future studies will be necessary to assess ZL0177’s activity on Nav1.1 and determine whether it differentially affects excitatory vs. inhibitory neuron subtypes.

The computational modeling simulations utilized in this study demonstrate the ability of ZL0177 to differentially modulate excitability in neurons gated by Nav1.6 or Nav1.2. In a biological system, however, direct suppression of Nav1.2 or Nav1.6-mediated currents may result in compensatory alterations to the expression or activity of other Nav isoforms [6], hindering the intended modulatory effect on excitability. Because HEK-Nav cell lines lack endogenous FGF14, the observed effects of ZL0177 reflect its direct action on Nav isoforms in the absence of FGF14. In native neurons, where FGF14 is highly expressed, the presence of this endogenous binding partner could influence compound access or binding affinity, and thus, the interaction dynamics may differ. This highlights the importance of future in vivo studies to explore ZL0177’s behavior in the physiological context of FGF14 expression. Despite this limitation, our results show that ZL0177 suppresses Nav1.2 and Nav1.6-mediated currents to a similar degree, while differentially modulating channel kinetics in an isoform-specific manner. This may be particularly useful in the case of Nav1.2, where functional deficiency induces hyperexcitability [17]. In support of this notion, investigations regarding the individual roles and effects of Nav1.2 and Nav1.6-specific pharmacological inhibition illustrate that dual blockade of Nav1.2 and Nav1.6 produces the most effective block of excess neuronal activity [6,68]. Thus, ZL0177 and compounds with similar activity profiles may provide the opportunity to modulate Nav1.2 channel kinetics while limiting excessive compensatory recruitment of Nav1.6; however, this possibility remains to be tested in systems where multiple Nav isoforms are endogenously expressed. Our NEURON simulations were based on parameters from individual cells, which ensured matched gating kinetics but may not fully reflect population averages. This limits the generalizability of the model and its predictions. Future work incorporating ensemble or averaged kinetic values could improve robustness.

Another limitation is the absence of Nav β-subunits in our expression system. While the α-subunit alone is sufficient to generate transient Na^+^ currents, β-subunits are known to modulate channel gating and drug responses. Thus, the activity of ZL0177 in native neurons may be influenced by α–β subunit interactions not captured in this system. For Nav1.6, we observed greater variability in the voltage dependence of inactivation under DMSO conditions. While statistical assumptions of normality and equal variance were met, this increased spread may reflect variability in patch seal quality or cell health that can occur even in standardized automated systems. Despite this, the directional trend of ZL0177’s lack of effect on Nav1.6 inactivation was consistent across replicates, supporting the robustness of the observation.

## Figures and Tables

**Table 1:** Summary of effects of 10 μM ZL0177 on Nav1.6 and Nav1.2 channel activity observed using the Synchropatch384i. Results are reported as mean ± SD.; significance was assessed using an unpaired *t*-test. ^a^ *p* = 0.0143; ^b^ *p* < 0.0001.

Na_v_ Isoform	Condition	Peak Current Density (pA/pF)
Na_v_1.2	DMSO	−228.3 ± 133.3 (22)
	ZL177	−130.7 ± 92.8 (17) ^a^
Na_v_1.6	DMSO	−51.2 ± 28.2 (17)
	ZL177	−21.6 ± 7.7 (20) ^b^

## Data Availability

The data that support the findings of this study are available from the corresponding author upon reasonable request.
